# Complete Genome Sequence of Bacillus cereus Bacteriophage vB_BceS_KLEB30-3S

**DOI:** 10.1128/MRA.00348-20

**Published:** 2020-05-14

**Authors:** Monika Šimoliūnienė, Deividas Tumėnas, Kotryna Kvederavičiūtė, Rolandas Meškys, Sigitas Šulčius, Eugenijus Šimoliūnas

**Affiliations:** a Department of Molecular Microbiology and Biotechnology, Institute of Biochemistry, Life Sciences Center, Vilnius University, Vilnius, Lithuania; b Department of Biological DNA Modification, Institute of Biotechnology, Life Sciences Center, Vilnius University, Vilnius, Lithuania; c Laboratory of Algology and Microbial Ecology, Nature Research Centre, Vilnius, Lithuania; DOE Joint Genome Institute

## Abstract

In this study, we present the genomic characterization of the temperate bacteriophage vB_BceS_KLEB30-3S (KLEB30-3S), which was induced from Bacillus cereus strain KR3M-30, isolated from a gypsum karst lake ecosystem in Lithuania. The 37,134-bp genome of KLEB30-3S contains 58 predicted protein-encoding genes and no tRNA genes.

## ANNOUNCEMENT

Bacillus cereus comprises a highly versatile group of bacteria which are of special interest because of their ecological significance, broad range of pathogenicity, and biocontrol potential (1, 2). A number of *Bacillus* phages and their components could be very useful tools for typing or detecting their hosts, significantly contribute to bacterial genetic diversity, and provide the host with special characteristics such as pathogenicity and distinct phenotypic traits (3–5). Thus, new isolation and insights into *Bacillus* phages remain highly relevant.

Here, we report the complete genome sequence of a temperate bacteriophage, vB_BceS_KLEB30-3S (KLEB30-3S), induced from Bacillus cereus strain KR3M-30 when cultured with KR3M-30-specific bacteriophage vB_BceS_KLEB30 (our unpublished data). The host strain was derived from a gypsum karst lake ecosystem in Biržai, Lithuania (56°14′55.5ʺN, 24°41′33.7ʺE), cultivated aerobically in LB at 22°C and identified using 16S rRNA gene sequence analysis. Phage KLEB30-3S was propagated using the soft-agar overlay method and purified using CsCl gradient ultracentrifugation as described previously (6). Phage DNA was isolated using the phenol-chloroform extraction and ethanol precipitation method (7). The complete genome sequence of KLEB30-3S was determined using Illumina DNA sequencing technology at BaseClear (Leiden, The Netherlands). Briefly, the sequencing library was prepared using in-house-developed library preparation solutions (BaseClear). Paired-end sequence reads were generated using the Illumina NovaSeq 6000 system (Illumina, San Diego, CA, USA). FASTQ read sequence files were generated using bcl2fastq2 version 2.18. Initial quality assessment was based on data passing the Illumina chastity filtering. Subsequently, reads containing the PhiX control signal were removed using an in-house filtering protocol (BaseClear). In addition, reads containing (partial) adapters were clipped (to a minimum read length of 50 bp). The second quality assessment was based on the remaining reads using the FastQC quality control tool version 0.11.5. The quality of the Illumina reads was improved using the error correction tool BayesHammer (8). Error-corrected reads were assembled into contigs using SPAdes version 3.10 (9). The contigs were linked together and placed into scaffolds using SSPACE version 2.3 (10). Using Illumina reads, gapped regions within scaffolds were (partially) closed using GapFiller version 1.10 (11). Finally, assembly errors and the nucleotide disagreements between the Illumina reads and scaffold sequences were corrected using Pilon version 1.21 (12).

In total, 168,858 high-quality reads were obtained and further assembled into one contig of 37,134 bp with an average coverage of 1,300-fold. The physical ends of the viral genome were identified using PhageTerm (13). Open reading frames (ORFs) were predicted with Geneious Prime 2019. The analysis of the genome sequence was performed using Transeq (http://www.ebi.ac.uk/Tools/st/emboss_transeq), Fasta-Nucleotide, Fasta-Protein, BLASTP, and Clustal Omega (http://www.ebi.ac.uk/Tools/msa/clustalo), as well as HHPred, HHblits, HMMER, and HHsenser (14). Also, tRNAscan-SE version 1.21 (http://lowelab.ucsc.edu/tRNAscan-SE) was used to search for tRNAs. The whole-genome comparative analysis using the online NCBI BLASTn (nucleotide collection [nr/nt] database; https://blast.ncbi.nlm.nih.gov) revealed that KLEB30-3S is closely related to the *Bacillus* bacteriophages from the genus *Lwoffvirus* (summarized in Table 1) (15–17). All bioinformatics tools described in this section were run with default parameters.

**TABLE 1:** Genomic characteristics of *Bacillus* phage KLEB30-3S and its closest relatives

Phage	GenBank accession no.	Genome size (bp)	G+C content (%)	No. of ORFs	Coverage with KLEB30-3S (%)	Identity with KLEB30-3S (%)
vB_BceS_KLEB30-3S	MT136606	37,134	38.3	58		
vB_BtS_BMBtp2	NC_019912	36,932	37.8	53	87	91.30
proCM3	KF_296717	43,278	37.4	66	87	90.56
TP21-L	NC_011645	37,456	37.8	56	83	90.19
BMBtpLA3	KX_190834	37,385	37.8	59	83	90.19

The genome of KLEB30-3S was predicted to be a linear, double-stranded, terminally redundant DNA molecule consisting of 37,134 bp. It has a G+C content of 38.3%, which is similar to that of Bacillus cereus (34.8 to 35.5%) (18). The genome of KLEB30-3S is closely packed with an average ORF size of 592 bp, and 91.82% of the genome is coding. It has 58 probable protein-encoding genes and no genes for tRNAs. While most of the KLEB30-3S genes were found to initiate from AUG (51 out of 58 ORFs), 4 ORFs were found to initiate with GUG and 3 with UUG. A marked asymmetry in the distribution of the genes on the two phage KLEB30-3S DNA strands was observed. The vast majority (56 out of 58) of KLEB30-3S ORFs have been predicted to be transcribed from the same DNA strand, whereas only two ORFs have been found on the opposite strand. Based on homology to biologically defined proteins, 19 ORFs of KLEB30-3S were given a putative functional annotation. The genome of KLEB30-3S appears to have a modular organization, with genes for DNA packaging (terminase small and large subunits), structure/morphogenesis (head morphogenesis protein, portal protein, scaffold protein, major capsid protein, head-tail connector, head-tail adaptor, tape measure protein, tail protein, and tail fiber protein), host lysis (holin and endolysin), lysogeny (integrase and repressor), and DNA replication/recombination (replicative DNA helicase and transcriptional regulators) clustered together. Notably, no virulence factors or antibiotic resistance determinants were detected in the genome of KLEB30-3S.

### Data availability.

The complete genome sequence of phage KLEB30-3S is available in the GenBank database under accession number MT136606. The accession number of the PCR-amplified 16S rRNA gene sequence of Bacillus cereus strain KR3M-30 is MN752435. The raw sequence reads are available in the SRA database under accession number SRR11441841 (BioProject number PRJNA613569 and BioSample number SAMN14409531).
